# MSCs ameliorate hepatocellular apoptosis mediated by PINK1-dependent mitophagy in liver ischemia/reperfusion injury through AMPKα activation

**DOI:** 10.1038/s41419-020-2424-1

**Published:** 2020-04-20

**Authors:** Jun Zheng, Liang Chen, Tongyu Lu, Yingcai Zhang, Xin Sui, Yang Li, Xuna Huang, Liying He, Jianye Cai, Chaorong Zhou, Jinliang Liang, Guihua Chen, Jia Yao, Yang Yang

**Affiliations:** 1 Department of Hepatic Surgery and Liver Transplantation Center of the Third Affiliated Hospital of Sun Yat-sen University, 510630 Guangzhou, China; 2 Guangdong Key Laboratory of Liver Disease Research, Key Laboratory of Liver Disease Biotherapy and Translational Medicine of Guangdong Higher Education Institutes, The Third Affiliated Hospital of Sun Yat-sen University, 510630 Guangzhou, China; 3 Surgical Intensive Care Unit, The Third Affiliated Hospital of Sun Yat-sen University, 510630 Guangzhou, China; 4 Central Experimental Room of the Third Affiliated Hospital of Sun Yat-sen University, 510630 Guangzhou, China; 5 Department of Gastroenterology, Guangzhou Women and Children’s Medical Center, 510630 Guangzhou, China

**Keywords:** Mitophagy, Apoptosis, Mesenchymal stem cells

## Abstract

Hepatocyte apoptosis is the main pathophysiological process underlying liver ischemia/reperfusion (I/R) injury. Mitochondrial abnormalities have a vital role in hepatocellular damage. The hepatoprotective effects of mesenchymal stem cells (MSCs) have been previously demonstrated. In this study, we aim to investigate the effect and potential mechanism of MSCs against liver I/R injury. Effects of MSCs were studied in mice liver I/R injury model and in a hypoxia/reoxygenation (H/R) model of L02 hepatocytes. The potential mechanisms of MSCs on these in vivo and in vitro I/R-induced hepatocellular apoptosis models were studies. Accompanied by the improvement of hepatic damage, MSCs exhibited capabilities of controlling mitochondrial quality, shown by reduced mitochondrial reactive oxygen species (mtROS) overproduction, decreased the accumulation of mitochondrial fragmentation, restored ATP generation and upregulated mitophagy. Furthermore, we descripted a potential mechanism of MSCs on upregulating mitophagy and found that the reduced Parkin and PINK1 expression and inactivated AMPKα pathway were observed in the liver tissue in I/R model. These effects were reversed by MSCs treatment. In vitro study showed that MSC-conditioned medium (MSC-CM) suppressed hepatocellular apoptosis and inhibited mtROS accumulation in the H/R environment. And these effects of MSC-CM were partially blocked after the cells were transfected with PINK1 siRNA or added with dorsomorphin. Collectively, our findings provide a novel pharmacological mechanism that MSCs exert hepatoprotective effect in liver I/R injury via upregulating PINK1-dependent mitophagy. In addition, this effect might be attributed to the modulation of AMPKα activation.

## Introduction

Liver ischemia/reperfusion (I/R) injury is one of the most important factors that induce liver dysfunction and even mortality during liver transplantation, major liver resection and hemorrhagic shock^1^. In response to mechanical hypoxic injury, ATP consumption and oxygen deletion induce increased hepatocyte death, whereas reperfusion induces high levels of inflammatory cell infiltration and reactive oxygen species (ROS) overproduction and thereby leads to extensive hepatocellular damage^2,3^. A further vicious cycle promotes development of liver injury, which has been attributed to interplay between inflammatory cascade and cell death. Therefore, identification of promising therapeutic strategies for targeting this pathophysiological process is necessary for alleviating liver I/R injury.

Mesenchymal stem cells (MSCs) have been gained attention as potential therapy for various diseases due to their capabilities of multipotent differentiation, regeneration and immunomodulation. Administration of MSCs appears to be a promising therapeutic strategy for I/R injury in brain, kidney and heart. Moreover, our and other recent studies have shown the role of MSCs in alleviating hepatic dysfunction in a liver I/R model^4–6^. However, detailed mechanism of hepatoprotective role of MSCs remains not fully understood.

Mitochondria are dynamic organelles that maintain cellular homeostasis and metabolic balance. Increasing evidence suggests that alterations in mitochondrial function contribute to hepatic pathology during I/R injury^7–13^. Mitochondrial damage results in excessive generation and accumulation of cellular ROS, which induce apoptosis by directly attacking cellular molecules and promoting inflammatory cells infiltration. Mitochondrial damage in response to I/R injury impacts energy metabolism of cells by reducing ATP generation and oxygen consumption during both ischemic and reperfusion phases, which negatively affects cell survival^14–16^. Hepatocytes, which contain an abundance of mitochondria, are sensitive to oxidative stress and thus vulnerable to mitochondrial injury. Therefore, recent investigations have focused on mitochondrial-target therapeutic strategies for liver diseases^17^.

Autophagy is a conserved cytoprotective process that maintains cellular homeostasis in vivo. This process involves degradation of long-lived proteins and dysfunctional organelles through formation of autophagosomes. Mitophagy, a type of autophagy, is an important process for maintenance of mitochondrial quality. It is regulated by PTEN-induced putative kinase 1 (PINK1) and Parkin, which accumulate on the mitochondrial membrane and stabilize mitochondrial depolarization^18^. Various experimental models of I/R injury have shown beneficial effect of hepatocellular mitophagy on cell survival^19,20^. Thus, therapeutic strategies for enhancing cellular mitophagy might constitute an efficient method for mitochondrial quality control and hepatoprotection during I/R injury.

In this study, the role of umbilical cord-derived MSCs (UC-MSCs) in attenuating hepatocellular damage in mice with I/R injury was investigated, and the mechanism underlying maintenance of mitochondrial quality and regulation of PINK1-dependent mitophagy through activation of AMPKα were elucidated. The mechanism through which UC-MSCs improve liver I/R injury indicates that these cells might constitute a therapeutic strategy for liver protection.

## Materials and methods

### Preparation of UC-MSCs

The preparation of UC-MSCs was approved by the Research Ethics Committee of the Third Affiliated Hospital of Sun Yat-sen University, and in this study, UC-MSCs were isolated and cultured as previously described^21^. All procedures were conducted using standardized protocols under aseptic condition. In brief, fresh umbilical cords (UCs) were collected from donors who provided informed consent and placed in phosphate-buffered saline (PBS) at 4 °C. Subsequently, the UCs were washed twice with PBS to remove remnant blood and cut into 10-mm^3^ pieces in type I collagenase with hyaluronidase (0.1%) containing CaCl_2_ (3 mM) and digested at 37 °C for 4 h. The specimens were cultured in low-sugar (1 g/l) Dulbecco’s modified Eagle’s medium (DMEM, Gibco, Life, Australia) containing 10% fetal bovine serum (FBS, PAN-Biotech, Germany) at 37 °C in a humidified atmosphere with 5% CO_2_. The medium was refreshed every 3 days to remove nonadherent cells, and the adherent cells were further cultured and passaged.

### Identification of the UC-MSC phenotype

The UC-MSCs were identified by analyzing the surface characteristics of the cultured cells through flow cytometry. In brief, the adherent cells were collected after the cells attained 70–80% confluence and washed with PBS containing 1% bovine serum albumin (BSA, Gibco, Life, Australia) to block nonspecific antigens. The cells were then incubated with monoclonal antibodies, including PE-CD105 (B76299), PE-CD73 (B68176), FITC-CD90 (IM1839U), FITC-CD45 (A07782), FITC-CD34 (IM1870) and RD1-CD29 (6604159) antibodies (Beckman), for 30 min according to the manufacturers’ instructions. The flow cytometry analysis was conducted using an eight-color FACSCalibur (BD Biosciences, NJ, USA), and the data were analyzed using FlowJo software (TreeSta, San Carlos, CA, USA).

### Multipotential differentiation of UC-MSCs

The potential of the UC-MSCs to differentiate into osteogenic and adipogenic cells was investigated. For osteogenic and adipogenic differentiation, UC-MSCs were cultured with specific osteogenesis medium and specific adipogenesis medium (Gibco, Life Technologies, NY, USA), respectively. The medium of each sample was replaced with fresh medium every 3 days. After 21 days of incubation, the ability of UC-MSCs to differentiate into osteogenic and adipogenic cells was assessed by Alizarin Red S and Oil Red O staining, respectively.

### Preparation of UC-MSC-conditioned medium

Conditioned medium (CM) from UC-MSCs (UC-MSC-CM) was obtained as previously described^5^. Briefly, for the preparation of UC-MSC-CM, the cells were grown to 70–80% confluence, washed twice with PBS and cultured in non-supplemented DMEM. The CM was collected after 48 h of incubation and centrifuged at 3200×*g* and 4 °C for 10 min.

### Animal management and establishment of hepatic ischemia–reperfusion injury model

Healthy, male C57BL/6 mice, aged 8–10 weeks and weighing 21–23 g, were purchased from the Guangdong Medical Laboratory Animal Center (Guangdong, China). The animal care procedures conformed to the Guidelines of Sun Yat-sen University for Animal Experimentation. All the mice were housed under a specific pathogen-free (SPF) room, given free access standard laboratory diet and water and maintained in a constant environment with a temperature of 20 °C, 50% humidity and a 12-h light/12-h dark cycle.

*Surgical procedure*: The animal experiments were performed following the National Institutes of Health Guide for the Care and Use of Laboratory Animals and approved by the Animals Care and Use Committee of Sun Yat-sen University (Guangzhou, China). The standard protocol for establishing a mouse 70% liver I/R injury model was described previously^22,23^. All surgical procedures were performed prior to anesthesia through an intraperitoneal injection of 0.6% pentobarbital sodium (100 μl/10 g). A midline laparotomy incision was performed. An atraumatic vascular clip was placed across the bile duct, portal vein and hepatic artery to interrupt 70% of the blood supplied to the liver, including the left lateral and median lobes of the liver. Ischemia was continued for 90 min and terminated by removing the atraumatic vascular clamp. The animals belonging to sham group underwent a midline laparotomy incision without any other operation.

*Group design*: The model mice were randomly divided into two groups (*n* = 5), namely, the PBS group and the UC-MSC group, and after reperfusion, the mice in these groups were administered 100 μl of PBS or UC-MSCs (10^6^/100 μl), respectively, through the peripheral vein and euthanized after 6 and 24 h of reperfusion. Liver and blood samples were collected for further experiments. To validate the mechanism of hepatocellular protection, four additional groups were established through the administration of 3-methyladenine (3-MA, IP; Sigma, Saint Louis, MO), UC-MSCs (10^6^/100 μl, i.v.) plus 3-MA (30 mg/kg, IP) or dorsomorphin (20 mg/kg, IP; Tocris, Bristol, UK), and MitoTEMPO (100 Nm, i.v.; Enzo Life Sciences, Inc., Farmingdale, NY, USA). After 6 h of reperfusion, the mice were killed for the collection of tissue and serum samples.

### Assessment of liver function

The levels of serum alanine aspartate aminotransferase (ALT), aspartate amiwnotransferase (AST) and lactate dehydrogenase (LDH) were detected using a 7180 Biochemical Analyzer (Hitachi, Japan). The degree of liver injury was also assessed based on the histological score.

### Histological analysis of liver tissue, immunohistochemistry (IHC) and immunofluorescence

Hematoxylin and eosin (H&E) staining was performed for the assessment of liver injury, and the histological score indicating the degree of liver injury was determined according to Suzike’s criteria^24^. The sections were observed under a light microscope by an observer who was blinded to the experimental groups, and five selected fields were randomly selected to obtain the injury score. Suzike’s criteria are listed in Supplemental Table 1.

To perform IHC staining, 4-μm-thick paraffin-embedded liver tissue slides were dewaxed, rehydrated, repaired and incubated with primary antibodies (caspase-3, PINK1, Parkin, LC3II and TOM20) overnight at 4 °C. The sections were then washed twice, incubated with the secondary antibody for 20 min at 37 °C and treated with diaminobenzidine. The sections were observed, and images were captured under a light microscope (Leica, Germany). Caspase-3 antibody (9662) was purchased from Cell Signaling Technology (USA), and PINK1 (ab23707), Parkin (ab77924), LC3II (ab51520) and TOM20 (ab186735) antibodies were purchased from Abcam (USA). Both the secondary antibody and diaminobenzidine were obtained from a kit (DAKO, USA).

### Evaluation of the superoxide dismutase (SOD) and malondialdehyde (MDA) contents

The MDA and SOD contents in liver tissues were detected using an MDA kit and a SOD kit (both kits were purchased from Nanjing Jiancheng Bioengineering Institute), respectively, according to the manufacturers’ instructions. The procedures are briefly described as follows:

1. SOD: The liver samples were mixed with the reagent and incubated at 37 °C for 20 min, and the absorbance at a wavelength of 450 nm was measured. The level of SOD activity and inhibition rate were calculated as follows: SOD activity (U/ml) = SOD inhibition rate/50% × dilution ratio in the reaction system/concentration of the sample protein to be tested; SOD inhibition rate = [(control A − blank control A) − (testing A − blank testing A)] × 100/(control A − blank control A).
2. MDA: The proteins were mixed with the reagent, incubated in a water bath at 95 °C for 40 min, cooled down in running water and centrifuged at 3500 rpm for 10 min. The MDA content was calculated as follows: MDA content (nmol/ml) = (absorbance of the testing tube − absorbance of the testing blank tube)/(the absorbance in the standard tube − the absorbance in the standard blank tube) × standard concentration (10 nmol/ml) × dilution ratio of the sample before the test. The absorbance values were obtained at a wavelength of 532 nm.

The absorbance of all the samples were detected using an automatic microplate reader (Biotek, Vermont, USA).

### Measure of apoptosis

The degree of apoptosis in the liver sections was detected through terminal deoxynucleotidyl transferase dUTP nick-end labeling (TUNEL) assay using a commercial in situ apoptosis detection kit (Roche Diagnostics, Indianapolis, IN, USA) according to the manufacturer’s instructions. In brief, the TUNEL reaction mixture was mixed with the enzyme and label solutions, and each section was incubated with the reaction mixture for 60 min in the dark at 37 °C. The sections were then washed twice with PBS and incubated with DAPI (KeyGEN BioTECH, Jiangsu, China) for 1 min at room temperature. Five fields were randomly selected from each section for assessment of the necrotic area, and the percentage of apoptotic cells was analyzed by an observer who was blinded to the group assignment. The sections were investigated and photographed under a fluorescence microscope (Leica, Germany), and the necrotic areas were evaluated using Image-Pro Plus 6.0 (USA).

The apoptosis of L02 cells was determined using a PI/Annexin V-FITC Apoptosis Detection Kit (BD, USA) according to the manufacturer’s recommended protocol. Briefly, L02 cells in each group were trypsinized, washed twice, and incubated for 15 min at room temperature in the dark. The degree of cell apoptosis was determined using a flow cytometer (BD Biosciences, NJ, USA).

### Protein extraction and western blotting

Liver tissues and L02 cells were lysed using cold radioimmunoprecipitation assay (RIPA) buffer containing 50 mM Tris-HCL (pH 7.4), 150 mM NaCl, 0.1% Triton X-100, 10% SDS, 10% sodium deoxycholate, 2 mM EDTA and protease cocktail inhibitor (Roche, Basel, Switzerland) for 20 min on ice. After the assessment of equal amounts of proteins, 30 μg of proteins was subjected to 12% SDS polyacrylamide gel electrophoresis (PAGE) and subsequently transferred onto polyvinylidene difluoride membranes (PVDF) (Millipore, Billerica, MA, USA). The membranes were incubated in 5% non-fat milk for 1 h and then with the primary antibodies, which included antibodies against Beclin1, Atg5, LC3, caspase-3, cleaved caspase-3, AMPKα (total), p-AMPKα, ULK1 (total), p-ULK1, PINK1, Parkin, TOM20, Bad and GAPDH, overnight at 4 °C. Then, the sections were incubated with the secondary antibody (anti-rabbit IgG, 1:5000, Sigma-Aldrich) for 1 h at room temperature. The blots were detected using an enhanced chemiluminescence (ECL) substrate and visualized using a FluorChem Systems imager (ProteinSimple, CA, USA). The intensities of the bands were analyzed by densitometric analysis using an image analyzer (ImageJ software, USA). AMPKα (5832), p-AMPKα (50081), ULK1 (8054), p-ULK1 (14202) and GAPDH (5174) antibodies were purchased from Cell Signaling Technology (USA), and Beclin1 (ab62557), Atg5 (ab108327), Bad (ab32445) was purchased from Abcam (USA).

### Transmission electron microscopy (TEM)

TEM was performed for the confirmation and monitoring of autophagy and the quantification of autophagic vacuoles. The liver samples were cut into 1-mm^3^ pieces and fixed with 2.5% glutaraldehyde in 0.1 M phosphate buffer at 4 °C for 1 h. After fixation and dehydration, ultrathin sections (70–80 nm) were obtained using an ultramicrotome (RMC MT6000-XL). All the sections were stained with lead citrate and uranyl acetate and detected under a transmission electron microscope (HT7700, HITACHI, Japan).

### Detection of ROS production in the liver tissues

The levels of ROS production in the liver tissues were determined by dihydroethidium (DHE) staining as previously described^25^. In brief, frozen sections of liver tissues in each group (5-μm-thick) were prepared and incubated with DHE (7.5 mM, MedChemExpress, China) for 30 min in the dark at 37 °C. After staining with DAPI, the sections were observed using a fluorescence microscope (Leica, Germany). Ten fields were randomly selected from each sample, and the percentage of DHE-positive cells was calculated through a quantitative morphometric analysis.

The levels of mtROS production in the liver tissues were measured by MitoSOX Red staining according to the manufacturer’s recommended protocol. Five-micron-thick frozen sections of liver tissues were incubated with MitoSOX Red (5 μM, Invitrogen) in the dark at 37 °C for 15 min. The sections were then stained with DAPI for 1 min in the dark and washed, and the number of MitoSOX Red-positive cells was counted as previously described^26^.

### Cell culture and establishment of hypoxia/reoxygenation (H/R) model

The human hepatocyte line L02 was purchased from Shanghai Cell Bank of the Chinese Academy of Sciences and was identified by STR profiling without mycoplasma contamination. The cells were cultured in high glucose-DMEM (4.5 g/l) with 10% PAN and maintained at 37 °C with 5% CO_2_. An H/R model of L02 cells was established for mimicking liver I/R injury as described previously^27^. Briefly, the cells were cultured under hypoxic conditions (94% N_2_, 5% CO_2_ and 1% O_2_) in a HERAcell 150i incubator (Thermo Fisher Scientific Company, UK) at 37 °C for 24 h and then transferred to the typical incubator (21% O_2_ and 5% CO_2_) for 4 h to allow reoxygenation.

*Group design*: This in vitro model was randomly divided into the following five groups: normal group, L02 cells were cultured in the typical medium without any intervention; H/R group, L02 cells were cultured in the typical medium after establishment of the H/R model; UC-MSCs-CM group, L02 cells were incubated under H/R conditions and treated with UC-MSCs-CM; UC-MSCs-CM+ dorsomorphin group, L02 cells were treated with UC-MSCs-CM in combination with dorsomorphin (2 μM) and then subjected to the H/R conditions (to explore the mechanisms of UC-MSCs in hepatoprotection); and UC-MSCs-CM+ PINK siRNA, L02 cells were pre-transfected with PINK1 siRNA, treated with UC-MSCs-CM and incubated in the H/R environment.

### Knockdown of PINK1 by siRNA

PINK1-related RNA interference was performed using the Lipofectamine® RNAiMAX reagent (Life, USA) and the most effective siRNA, Hs_PINK1_6 FlexiTube siRNA NM032409 (sequence: ATGGGTCAGCACGTTCAGTTA), to knockdown gene and protein expression in L02 cells. In addition, L02 cells transfected with non-silencing scrambled siRNA were used as a control group.

### Assessment of mtROS in L02 cells

The mtROS in L02 cells was detected using MitoSOX Red (Thermos Fisher Scientific, M36008) according to the manufacturer’s instructions. In brief, the groups were subjected to their specific treatments, washed twice with PBS and then incubated with MitoSOX Red (5 μM) for 10 min at 37 °C in the dark. The samples were then washed three times, and the mtROS of the cells were determined through flow cytometry analysis using an eight-color FACSCalibur.

### Detection of intracellular ATP concentration

The concentration of ATP in the L02 cells belonging to the different groups was measured using an ENLITEN ATP assay system bioluminescence detection kit (Promega, USA) following the manufacturer’s recommended protocol.

### Detection of the mitochondrial membrane potential

The mitochondrial electrochemical gradient (△Ψm) of the inner mitochondrial membrane of the L02 cells was detected through a flow cytometric analysis of the cationic dye MitoProbe (5′,6,6′-tetrachloro-1,1′,3,3-tetraethylbenzimidazol-carbocyanine iodide, JC-1, Life Technologies) according to the manufacturer’s instructions. In brief, L02 cells were trypsinized, suspended in 2 ml of pre-warmed PBS containing 2 μl of JC-1, and incubated for 30 min in the dark at 37 °C. After three washes with PBS, the JC-1 fluorescence of the L02 cells was measured using an eight-color FACSCalibur.

### Quantification of mitochondrial DNA (mtDNA)

The mtDNA from L02 cells was extracted using a Mito DNA Extraction Kit (Genmed Scientifics Inc., USA) in accordance with the manufacturer’s recommended protocol and amplified by qRT-PCR using a SYBR Green detection system (Roche Applied Science, Mannheim, Germany) and a thermocycler (LightCycler® 96-Wells, Roche Applied Science) to measure the amount of mtDNA in the cytosol. The mtDNA was probed using primers for the specific detection of Complex II (succinate-ubiquinone oxidoreductase): forward, 5′-CAAACCTACGCCAAAATCCA-3′, and reverse, 5′-GAAATGAATGAGCCTACAGA-3′. For normalization, the following GAPDH primers were used: forward, 5′-CTGACTTCAACAGCGACACC-3′, and reverse, 5′-GTGGTCCAGGGGTCTTACTC-3′.

### Immunofluorescence

For the assessment of cell immunofluorescence, L02 cells from each group were fixed with 4% paraformaldehyde for 30 min and blocked with 5% BSA for 1 h. The cells were then incubated with primary antibodies (TOM20 and LC3II, 1:200) overnight at 4 °C and then with Alexa Flour 488- and 594-conjugated secondary antibodies (Invitrogen) for 1 h in the dark at 37 °C. The cell nuclei were then stained with DAPI for 1 min at room temperature in the dark. The stained L02 cells were investigated and photographed under a confocal microscope (LSM710, Zeiss, German).

### Statistical analysis

The data are shown as the mean ± SD or as direct values. All the statistical analyses were performed using Prism statistical software (GraphPad) version 5.0 (USA). The comparisons of two groups were analyzed using Student’s two-tailed *t*-test, and the comparisons of three or more groups were assessed by one-way ANOVA. A probability (*p*) value of <0.05 or <0.01 was considered to indicate a significant difference.

## Results

### UC-MSCs attenuate hepatocellular damage and upregulate autophagy in the liver I/R injury model

**Fig. 1:**
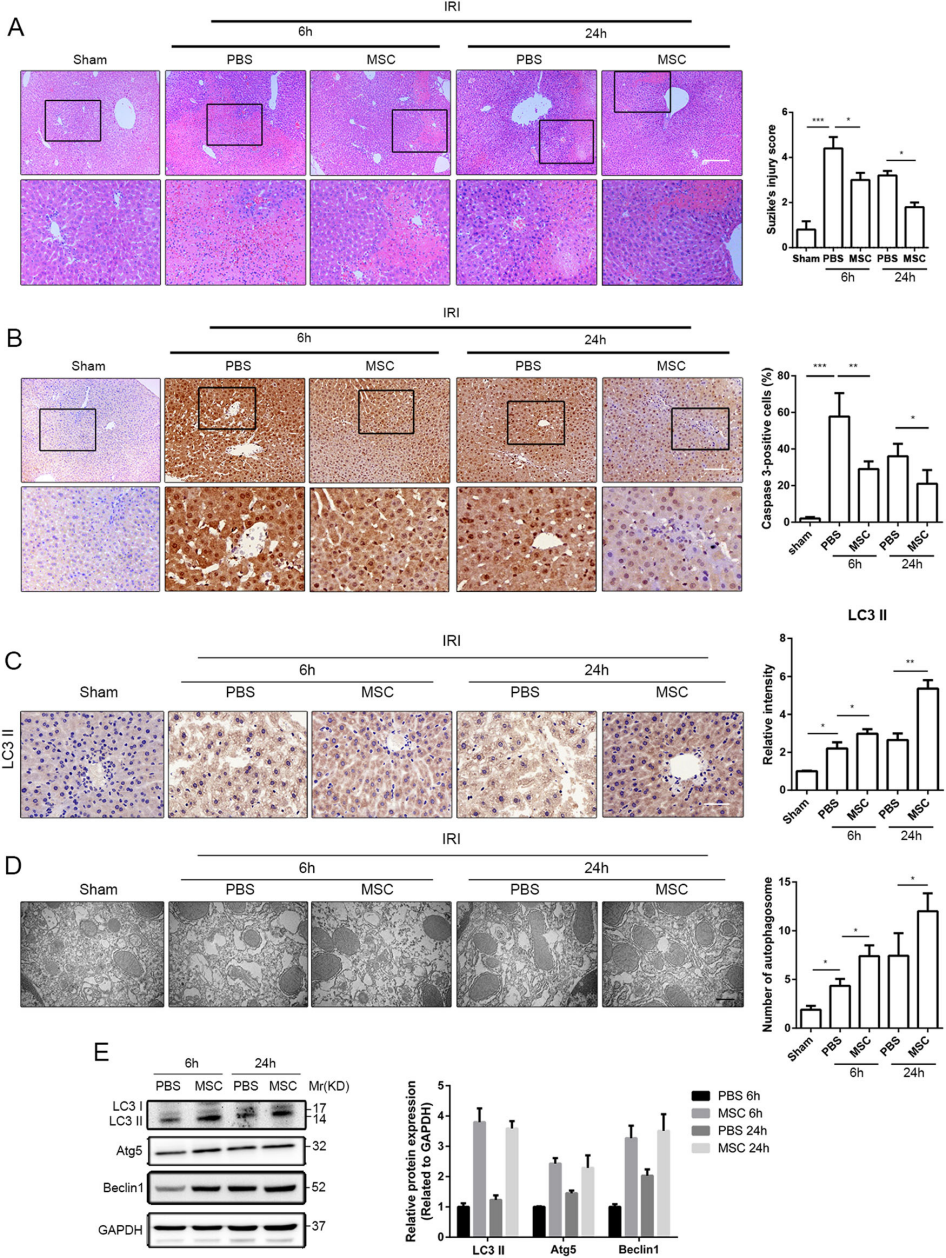
Effects of UC-MSC treatment on attenuating injury and upregulating autophagy in the liver of mice with I/R injury. **a** Representative histological sections of livers stained with hematoxylin and eosin were obtained (H&E staining, magnification ×100). Livers from each experimental group were subjected to histological evaluation, and the degree of liver injury was determined according to Suzike’s injury score (*n* = 5). **b** Representative liver sections from each group stained with caspase-3 were obtained (magnification ×200). The IHC staining of caspase-3 in liver tissues was semiquantified (*n* = 5). **c** Representative liver sections from each group stained with LC3II were obtained (magnification ×400). The IHC staining of LC3II in liver tissues was semiquantified (*n* = 5). **d** Representative TEM images were obtained for the measurement of autophagic vacuoles in liver tissues from each experimental group. The count of autophagic vacuoles in the livers was measured (*n* = 5). **e** The autophagy-related proteins in liver tissues obtained from mice with I/R injury after 6 and 24 h of reperfusion and treated with PBS or UC-MSCs were assessed by a western blotting assay. The average intensities of the band in the western blots were quantified using GAPDH as an internal reference. All the data are shown as the means ± SEMs (*n* = 5/group). *p < 0.05, **p < 0.01, ***p < 0.001 (all *p*-values were obtained by one-way ANOVA).

Human UC-MSCs were successfully isolated, and the characteristics of UC-MSCs were shown in Supplemental Fig. 1A, B. After 6 and 24 h of reperfusion, the liver I/R model mice showed severely impaired liver function (Supplemental Fig. 2A) and obvious hepatocellular injury, as demonstrated by increased infiltration of inflammatory cells, disorder of hepatic lobules and tissue necrosis (Fig. 1a). The intravenous transfusion of UC-MSCs after reperfusion exhibited a hepatoprotective role, as shown by decreasing ALT, AST and LDH levels (Supplemental Fig. 2A), and a reduction in Suzike’s score (Fig. 1a). Furthermore, activity of caspase-3 was identified as an indicator for the severity of hepatocellular apoptosis. As shown in Fig. 1b, transplanted UC-MSCs significantly decreased the expression of caspase-3 in liver tissue after 6 and 24 h of reperfusion. Western blot assays revealed similar expression levels of caspase-3 and apoptosis-related protein (Bad) (Supplemental Fig. 1B). TUNEL assay also demonstrated that UC-MSCs had an important role in inhibiting hepatocellular apoptosis after I/R injury (Supplemental Fig. 1C). In addition, the IHC staining of the LC3, a widely used marker for autophagy, showed that I/R upregulated the expression of LC3 in hepatocytes and that this upregulation was significantly enhanced by UC-MSCs (Fig. 1c). TEM micrographs of liver tissues from I/R mice revealed that compared with the control group, the number of autophagosomes in the liver tissues was substantially increased after UC-MSC treatment (Fig. 1d). Furthermore, it is well known that conversion of the soluble form of LC3 (LC3I) to autophagic membrane-associated form (LC3II) represents autophagosome formation. In addition, the other autophagy-related proteins, Beclin1 and ATG5, are the first mammalian genes that are positively associated with autophagy and important for autophagosome formation. Thus, the LC3II/I ratio and the expression of Beclin1 and ATG5 are used for detection of autophagic levels. Results of western blot assays revealed that 6 and 24 h after reperfusion, administration of UC-MSCs significantly increased the LC3II/I ratio and notably elevated the expression of both Atg5 and Beclin1 (Fig. 1e).

### UC-MSCs attenuated oxidative stress in the liver of mice with I/R injury

**Fig. 2:**
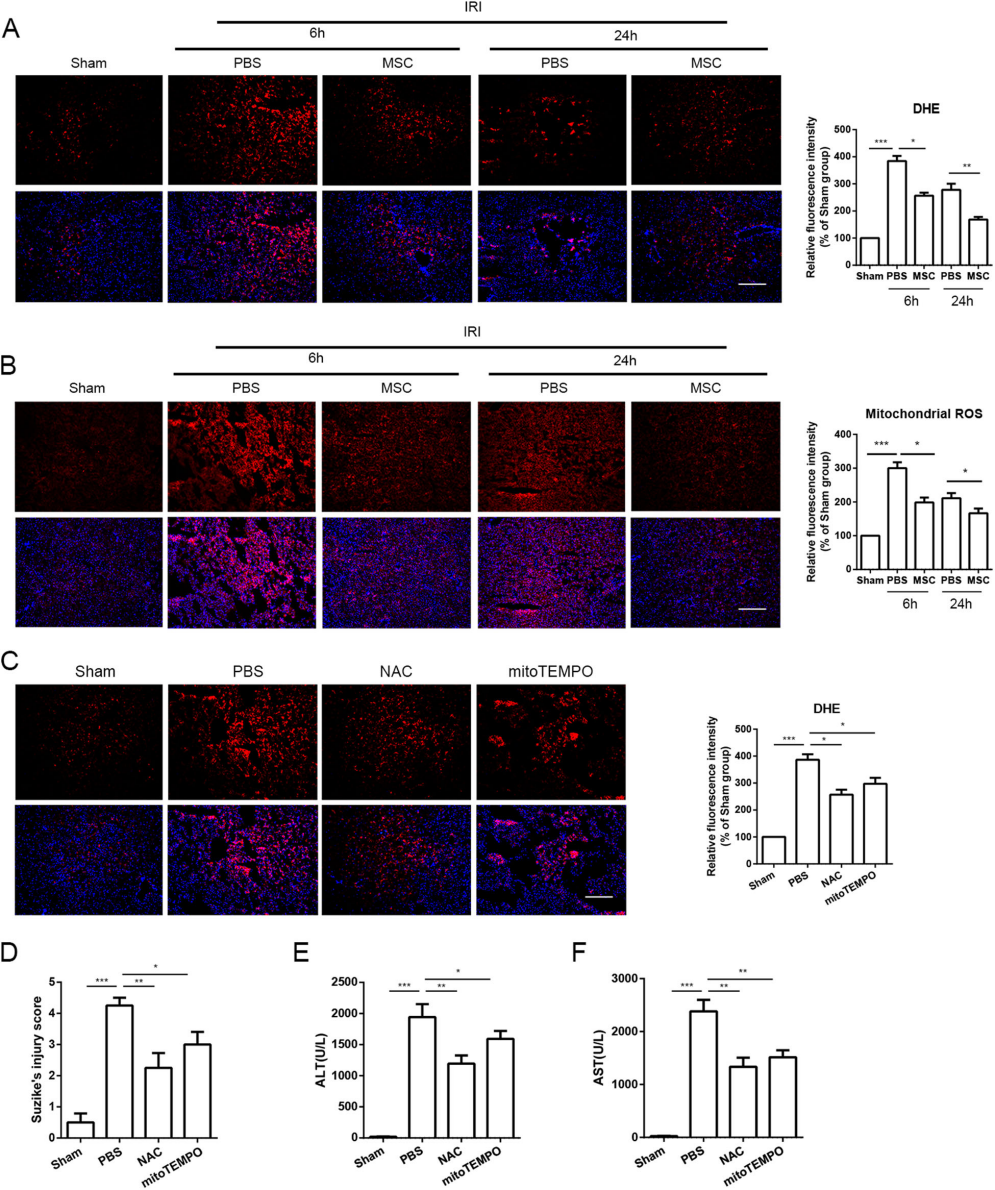
Effects of UC-MSC treatment on inhibiting the generation and distribution of ROS and mtROS in the liver of mice with I/R injury. Mice that underwent hepatic I/R injury and were treated with UC-MSCs or PBS were killed at 6 and 24 h after reperfusion. **a** Representative liver sections from each group stained with DHE (red) and DAPI (blue) were obtained to determine intracellular ROS production (magnification ×100). The data are expressed as the means ± SEMs (*n* = 5/group). **b** Representative liver sections stained with MitoSOX Red (red) and DAPI (blue) were obtained for the determination of mtROS production in liver tissues (magnification ×100). The data are expressed as the means ± SEMs (*n* = 5/group). **c** To confirm that mtROS have a key role in the induction of liver injury after I/R, we included an additional group, in which Mito-TEMPO, a scavenger of mtROS, was used for the treatment of liver I/R injury. The mice were killed 6 h after reperfusion. Representative liver sections stained with DHE and DAPI showed that the fluorescent density of DHE was significantly decreased after Mito-Tempo administration compared with that obtained after PBS treatment (magnification ×100). The values are presented as the means ± SEMs (*n* = 5/group). **d** After H&E staining, the degree of liver injury in each group was detected according to Suzike’s injury score. The data are expressed as the means ± SEMs (*n* = 5/group). **e**, **f** Treatment with Mito-Tempo attenuated the levels of ALT and AST compared with the levels found in the PBS group. The data are expressed as the means ± SEMs (*n* = 5/group). *p < 0.05, **p < 0.01, ***p < 0.001 (all *p*-values were obtained by one-way ANOVA).

As shown in Fig. 2, we detected changes in oxidative stress, including generation and distribution of ROS, in liver tissues after I/R injury in the presence or absence of UC-MSCs. DHE is used to measure the level of generalized oxidative stress, which is oxidized by O_2_^−^28. As shown in Fig. 2a, I/R injury induced a significant increase in DHE oxidation to 2-hydroxyethidium (red fluorescence), which indicates increase in generation and distribution of ROS (O_2_^−·^) in liver tissues. In addition, treatment to liver I/R injury with UC-MSCs in mice reversed these effects by inhibiting DHE oxidization (Fig. 2a). We also detected levels of MDA and SOD in liver tissues to further confirm the severity of oxidative stress in each group, and UC-MSCs treatment group showed increased SOD activity and decreased MDA content compared with untreated group (Supplemental Fig. 3A, B). Mitochondrial ROS (mtROS) constitutes one of the main forms of ROS. We subsequently used MitoSOX Red, a mitochondrial superoxide indicator, to investigate whether I/R-induced ROS were derived from mitochondria. Compared with sham group, MitoSOX Red fluorescence was significantly increased in the liver tissues after I/R injury, indicating I/R-induced ROS formation in mitochondria. UC-MSCs significantly inhibited mtROS formation, as shown by reduction in the red fluorescence intensity in liver tissues (Fig. 2b).

We further detected the effects of ROS on I/R-induced liver injury through the administration of NAC and Mito-TEMPO, which are scavengers of total ROS and mtROS, respectively. The results suggested that NAC and Mito-TEMPO not only effectively inhibited increase in ROS induced by I/R injury (Fig. 2c) but also concomitantly attenuated injury to the liver, as evidenced by improvements in liver function, pathological injury and severity of hepatocellular apoptosis (Fig. 2d–f).

### 3-MA reversed the hepatoprotective effect of UC-MSCs in mice with liver I/R injury

**Fig. 3:**
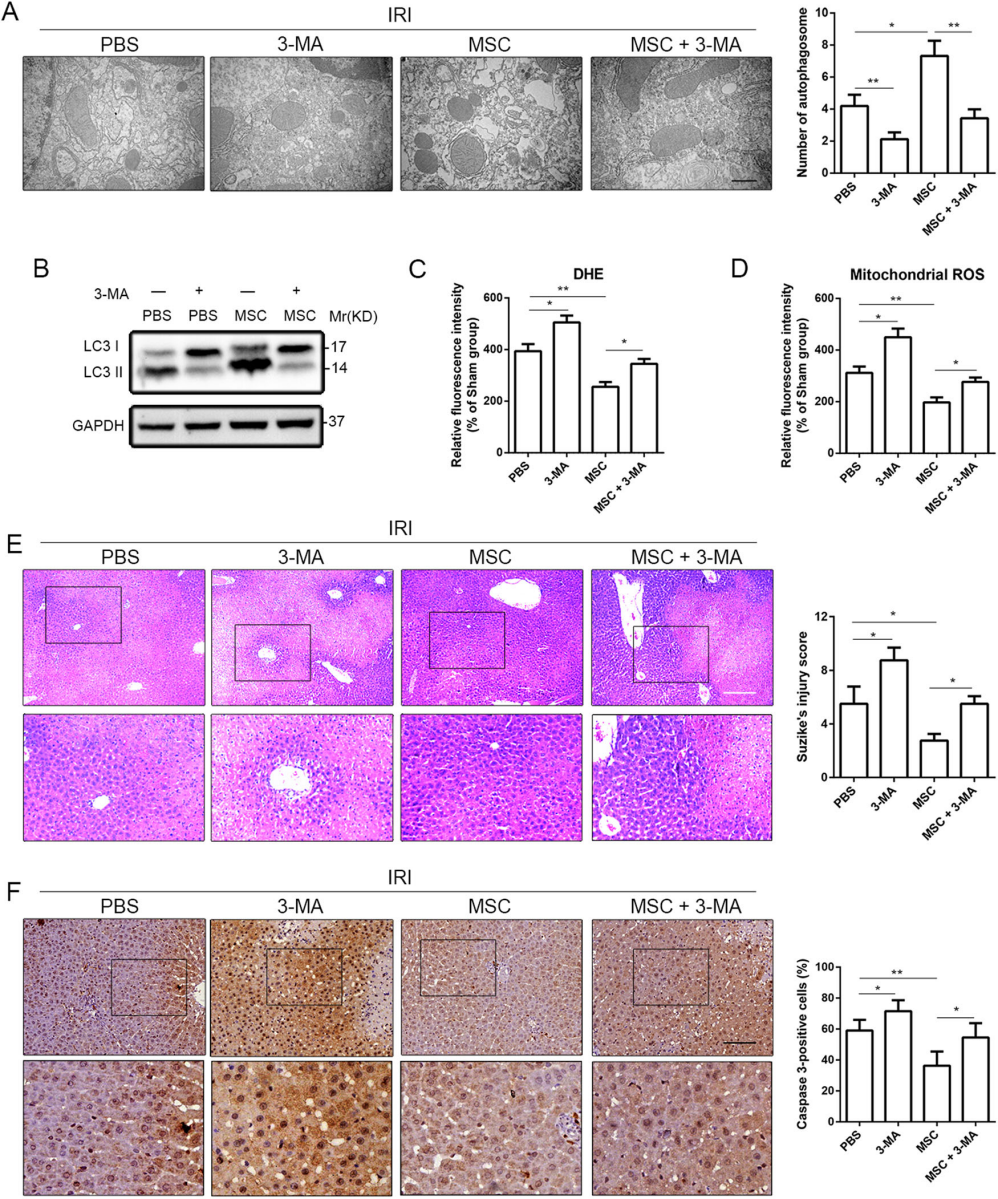
3-MA weakens the effects of UC-MSC treatment on hepatoprotection and upregulation of autophagy in the liver of mice with I/R injury. Mice with liver I/R injury were treated with PBS, 3-MA, UC-MSCs or UC-MSCs+3-MA were killed 6 h after reperfusion. **a** The number of autophagic vacuoles was determined by TEM. The data are presented as the means ± SEMs (*n* = 5/group). **b** Western blot assays were performed to detect the level of autophagy in liver tissues. **c** Representative liver sections from each experimental group stained with DHE and DAPI were obtained to determine the total ROS level. The data are presented as the means ± SEMs (*n* = 5/group). **d** Representative liver sections from each experimental group stained with MitoSOX Red and DAPI were obtained to determine the level of mtROS. The data are presented as the means ± SEMs (*n* = 5/group). **e** H&E staining was used for the assessment of liver injury (magnification ×100). The data are presented as the means ± SEMs (*n* = 5/group). **f** Representative liver sections from each group stained with caspase-3 were obtained (magnification ×200). The IHC staining of caspase-3 in liver tissues was semiquantified (*n* = 5). *p < 0.05, **p < 0.01, ***p < 0.001 (all *p*-values were obtained by one-way ANOVA).

To confirm the involvement of autophagy in ROS generation and protective effect of UC-MSCs on liver I/R injury, we treated liver I/R injury model mice with both 3-methyladenine (3-MA), an autophagy antagonist, and UC-MSCs and detected the resulting change in liver injury and hepatocellular apoptosis. TEM results showed that simultaneous administration of UC-MSCs and 3-MA substantially decreased the number of autophagic vacuoles compared with the group treated with UC-MSCs alone (Fig. 3a). As expected, western blot to evaluate LC3II/I ratio yielded similar results (Fig. 3b). In addition, 3-MA blocked the alleviative effect of UC-MSCs on oxidative stress (total ROS and mtROS) in liver tissue after I/R (Fig. 3c, d). We subsequently investigated the effect of 3-MA on abolishing the protective role of UC-MSCs. Addition of 3-MA reversed improvement in serum levels of hepatic enzymes (ALT and AST) (Supplemental Fig. 4A) and pathological changes in liver architecture (H&E staining and Suzike’s score) (Fig. 3e). Furthermore, as shown above, we found that UC-MSCs inhibited hepatocellular apoptosis during I/R injury. However, IHC staining and western blotting revealed that 3-MA upregulated the UC-MSC-reduced expression of caspase-3 (Fig. 3f and Supplemental Fig. 4B, C, respectively) and the number of TUNEL-positive cells (Supplemental Fig. 4D, E), indicating that the role of UC-MSCs in inhibiting hepatocellular apoptosis during I/R injury was weakened by treatment with 3-MA. We added a liver I/R injury group that was treated with 3-MA alone to clarify the hepatoprotective effect of autophagy, and the results showed that the 3-MA-mediated decrease in autophagy (Fig. 3a, b) was accompanied by aggravated liver injury (Fig. 3c–f and Supplemental Fig. 4a–e), which is consistent with previous studies^29,30^.

### UC-MSCs upregulated PINK1-dependent mitophagy in hepatocytes of mice with I/R injury

**Fig. 4:**
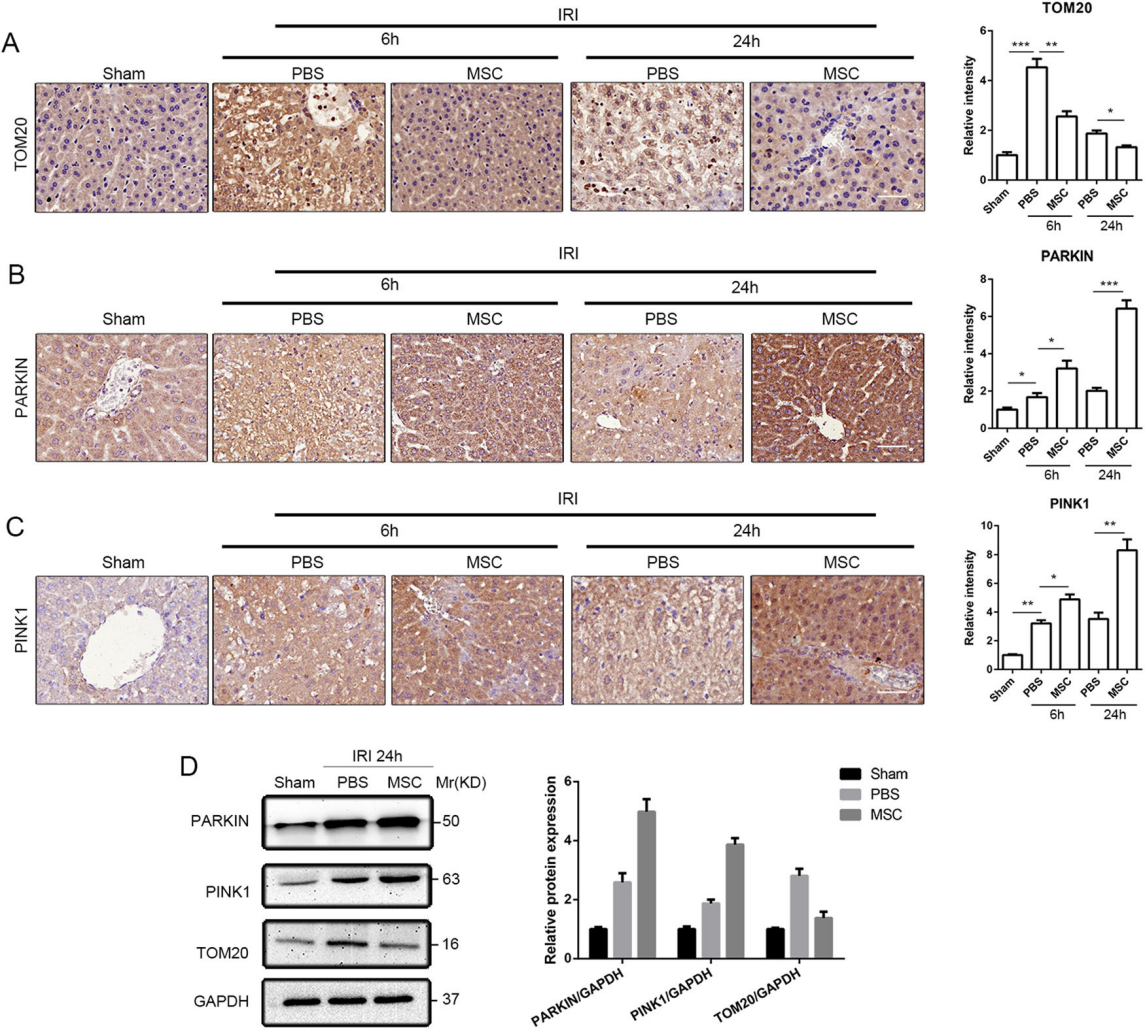
Effects of UC-MSCs on regulating the expression of mitophagy-related proteins in the liver of mice with I/R injury. **a**–**c** IHC of liver sections (magnification ×400) from mice with I/R injury treated with UC-MSCs or PBS and killed at 6 and 24 h after reperfusion were stained with TOM20 (column 1), Parkin (column 2) and PINK1 (column 3) antibody. The IHC staining of PINK1, Parkin and TOM20 in liver tissues was semiquantified (*n* = 5/group). **d** The expression of Parkin, PINK1 and TOM20 in liver tissues was determined by western blotting, and the average intensities of the bands in the western blot were quantified (*n* = 5/group). The data are presented as the means ± SEMs. *p < 0.05, **p < 0.01, ***p < 0.001 (all *p*-values were obtained by one-way ANOVA).

TOM20 is widely used to monitor the mitochondrial mass during mitophagy, and a decreased expression of TOM20 is considered to indicate a reduced mitochondrial mass and upregulated mitophagy. IHC staining of TOM20 revealed a significant increase in TOM20 expression in hepatocytes of mice with liver I/R injury compared with the sham group, and UC-MSCs treatment notably reversed this change (Fig. 4a). We also detected the mitophagy-associated proteins PINK1 and Parkin through IHC staining to explore the mechanism underlying the role of UC-MSCs in inducing mitophagy in hepatocytes of mice with liver I/R injury. As shown in Fig. 4b, c, treatment with UC-MSCs increased the expression of PINK1 and Parkin in liver tissues from mice with I/R injury compared with PBS group. Western blot assays were subsequently performed to further confirm the above-mentioned results, and similar changes was found in protein expression of PINK1, Parkin and TOM20 (Fig. 4d).

### The effect of UC-MSCs on hepatoprotection in I/R injury mice was dependent on AMPKα activation

**Fig. 5:**
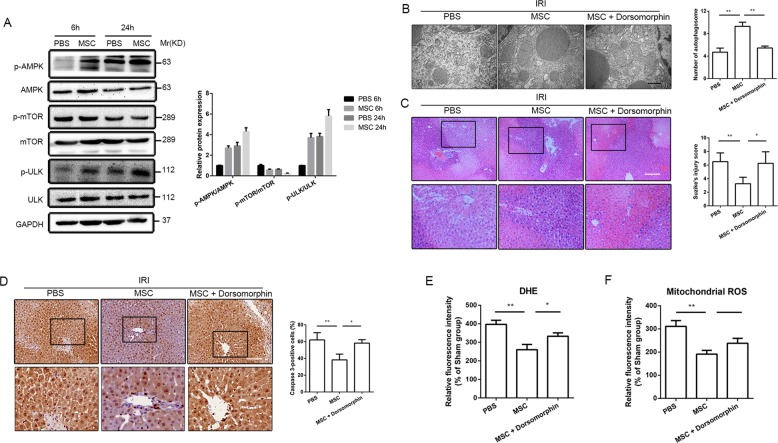
The effects of UC-MSCs on upregulating autophagy in the livers of mice with I/R injury depends on the activation of AMPKα. To detect the activation of AMPK and its downstream autophagy-related signaling pathway, mice with liver I/R injury and treated with PBS or UC-MSCs were killed at 6 and 24 h after reperfusion. In addition, to investigate the role of AMPK activation, model mice treated with UC-MSCs and dorsomorphin were killed 6 h after reperfusion. **a** Western blot analysis revealed the expression of AMPK, p-AMPK, mTOR, p-mTOR, ULK and p-ULK in liver tissues from each group. The average intensities of the band in the western blots were quantified using GAPDH as an internal reference. **b** TEM was used to determine the number of autophagic vacuoles. The data are presented as the means ± SEMs (*n* = 5/group). **c** The hepatic histology of each experimental group was obtained by H&E staining (magnification ×100), and the degree of liver injury was determined using Suzike’s injury score. The data are presented as the means ± SEMs (*n* = 5/group). **d** Representative liver sections from each group stained with caspase-3 were obtained (magnification ×200). The IHC staining of caspase-3 in liver tissues was semiquantified (*n* = 5). The data are presented as the means ± SEMs (*n* = 5/group). **e** Representative liver sections from each experimental group stained with DHE and DAPI were obtained to determine the level of total ROS. The data are presented as the means ± SEMs (*n* = 5/group). **f** Representative liver sections from each experimental group stained with MitoSOX Red and DAPI were obtained to determine the level of mtROS. The data are presented as the means ± SEMs (*n* = 5/group). *p < 0.05, **p < 0.01, ***p < 0.001 (all *p*-values were obtained by one-way ANOVA).

We further detected potential molecular mechanism underlying the role of UC-MSCs on hepatocyte PINK1-dependent mitophagy in mice with I/R injury. Previous studies showed AMPKα is associated with PINK1-dependent mitophagy^30,31^. Thus, in the present study, we investigated whether the induction of hepatocellular PINK1-dependent mitophagy by UC-MSCs in I/R mice was dependent on AMPKα activation. We first performed western blot assays to detect the levels of phosphorylated AMPKα in the liver tissues at 6 and 24 h after reperfusion. The results showed that administration of UC-MSCs significantly increased the levels of AMPKα phosphorylated at threonine 172 in the liver tissues compared with the control group (Fig. 5a). Subsequently, we measured the level of autophagy-related phosphorylated proteins downstream of AMPKα and found that the level of mTOR at p70S6K, a key protein that serves as a negative indicator of autophagy, was notably lower in the UC-MSCs group than that in the control group. In contrast, in addition to AMPKα activation, level of phosphorylated ULK1 at Ser555 was also significantly elevated in liver tissue in UC-MSCs group (Fig. 5a). To determine whether the hepatoprotective effects of UC-MSCs depend on AMPKα activation, we used a specific AMPK antagonist, dorsomorphin, to inhibit AMPK activation and investigated the therapeutic effects of UC-MSCs in mice with liver I/R injury. As shown in Fig. 5b, upregulated autophagy of UC-MSCs was notably blocked by dorsomorphin, as confirmed by TEM. We also detected the expression of Parkin and PINK1 in liver tissue through western blot assays. The results showed that dorsomorphin significantly reduced the UC-MSC-mediated increase in the expression of Parkin and PINK1 (Supplemental Fig. 5A). The combined treatment with dorsomorphin and UC-MSCs showed that dorsomorphin not only revered the effect of UC-MSCs on enhancing autophagy in hepatocytes but also weakened the hepatoprotective effect of UC-MSCs after I/R injury, as demonstrated by reversal of the reduction in the level of serum liver enzymes (Supplemental Fig. 5B) and improvement in hepatic pathology (Fig. 5c). The anti-apoptosis effect of UC-MSCs was also abrogated by dorsomorphin, as indicated by increase of both caspase-3 expression (Fig. 5d) and number of TUNEL-positive cells (Supplemental Fig. 5C) in the liver tissues compared with group treated with only UC-MSCs. Furthermore, we also found that dorsomorphin affected the role of oxidative stress in UC-MSCs. The DHE staining assay demonstrated that the blockage of AMPKα significantly abolished the UC-MSC-reduced generation of O_2_^−·^ in the liver (Fig. 5e). As demonstrated by MitoSOX Red staining, level of mtROS in liver tissues from UC-MSC-treated mice with liver I/R injury was notably increased after inhibited AMPKα activation (Fig. 5f).

### UC-MSC-CM maintained the mitochondrial quality and inhibited hepatocellular apoptosis through the induction of mitophagy

**Fig. 6:**
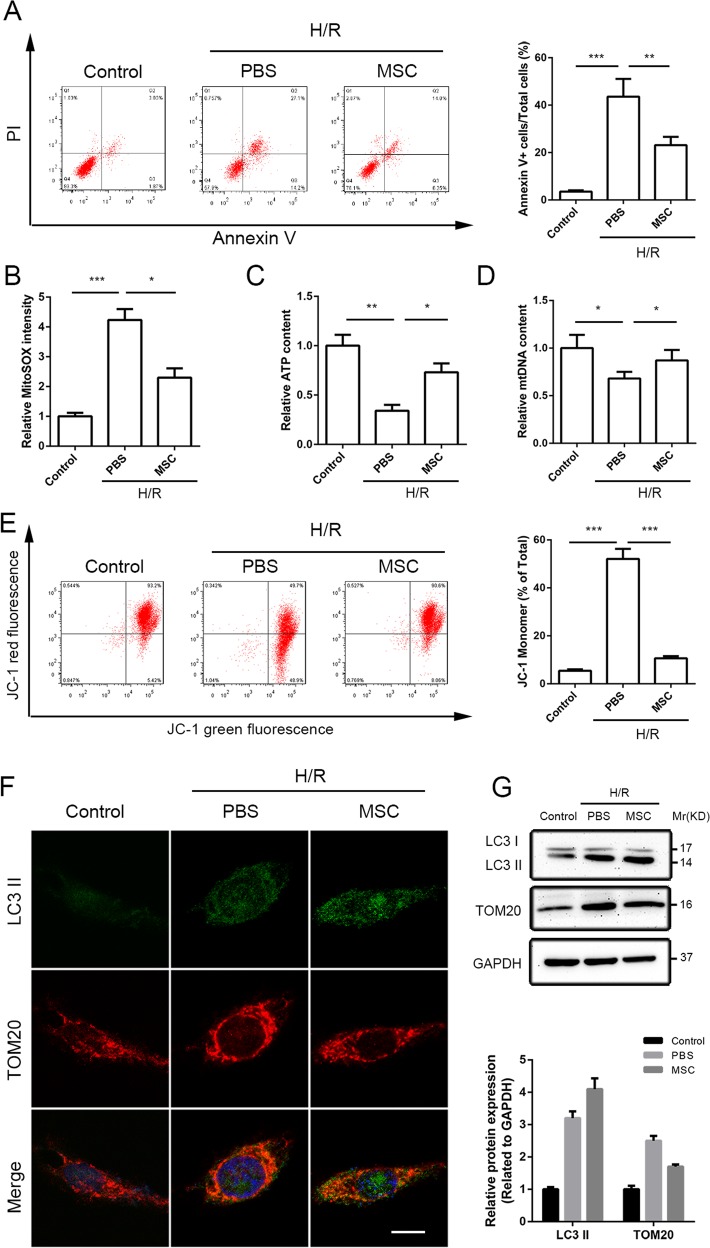
UC-MSC-CM protects L02 hepatocytes and induces mitophagy in L02 cells exposed to H/R conditions. L02 cells were respectively treated with normal cell culture medium, PBS or UC-MSC-CM and exposed to H/R conditions. **a** Cell apoptosis was determined through the flow cytometric detection of PI/Annexin V. The data are presented as the means ± SEMs (*n* = 3/group). **b** The level of mtROS generation in each experimental group was detected by MitoSOX Red staining and analyzed by flow cytometry. The data are presented as the means ± SEMs (*n* = 3/group). **c** The level of ATP production in each group was measured. The data are presented as the means ± SEMs (*n* = 3/group). **d** For the assessment of mitochondrial injury, the level of mtDNA in L02 cells in each group was detected. The data are presented as the means ± SEMs (*n* = 3/group). **e** The △Ψm of the inner mitochondrial membrane of L02 cells in each group was detected through the flow cytometric assessment of JC-1 staining. The data are presented as the means ± SEMs (*n* = 3/group). **f** The coimmunofluorescence of LC3II (fluorescent green) and TOM20 (fluorescent red) was assessed to measure the level of mitophagy in L02 cells in each group. Scale bar: 10μm. **g** The expression of TOM20 and the LC3II/I ratio in L02 cells in the various groups were determined by western blot assay. The average intensities of the band in the western blots were quantified using GAPDH as an internal reference. The data are presented as the means ± SEMs (*n* = 3). *p < 0.05, **p < 0.01, ***p < 0.001 (all *p*-values were obtained by one-way ANOVA).

We established an in vitro H/R model using L02 hepatocytes to mimic liver I/R injury to further explain the hepatic protective potential of UC-MSCs on liver I/R injury. The UC-MSC-CM-mediated activation of mitophagy was investigated by focusing on mitochondrial quality, which is the attack target of I/R injury. First, flow cytometric results showed that H/R injury notably increased apoptosis rate of hepatocytes compared with the control group, whereas UC-MSC-CM attenuated cellular apoptosis under the H/R conditions (Fig. 6a).

The main function of mitochondria involves maintenance of energy balance and participation in the process of cell metabolism. The mitochondrial quality was detected by investigating levels of mtROS production in the three groups through MitoSOX staining. Flow cytometric results demonstrated that H/R treatment significantly increased the mtROS level in primary cultured hepatocytes, whereas UC-MSC-CM blocked the production of mtROS (Fig. 6b). Moreover, the ATP level was robustly decreased in the hepatocytes treated with H/R, and UC-MSC-CM reversed this effect (Fig. 6c). In addition, mtDNA was detected by the mt-Atp6:Rpl13 ratio, which is an indicator used to indirectly assess the mitochondrial amount^32^. The results showed that exposure to H/R conditions increased the number of mitochondrial fragments in the hepatocytes compared with control group, whereas UC-MSC-CM reduced the amount of mitochondria (Fig. 6d). In addition, compared with the control group, the △Ψm was notably unbalanced in L02 cells after treatment with H/R, but this effect was significantly reversed by addition of UC-MSC-CM (Fig. 6e).

To monitor mitophagy in hepatocytes, mitochondrial fragments were observed by TOM20 staining (red fluorescence), and autophagosomes were visualized based on expression level of LC3 (green fluorescence). UC-MSC-CM significantly enhanced the overlap between autophagosomes and mitochondria in hepatocytes exposed to H/R compared with that found in the untreated hepatocytes (Fig. 6f). We also detected the protein levels of LC3II after UC-MSC-CM treatment in this model and found that UC-MSC-CM clearly increased the LC3II/I ratio in hepatocytes (Fig. 6g). Conversely, expression of TOM20 was significantly increased in the PBS group and decreased after UC-MSC-CM treatment (Fig. 6g).

### PINK1-dependent mitophagy inhibition blocked the UC-MSC-CM-mediated hepatocellular protection during exposure to H/R conditions

**Fig. 7:**
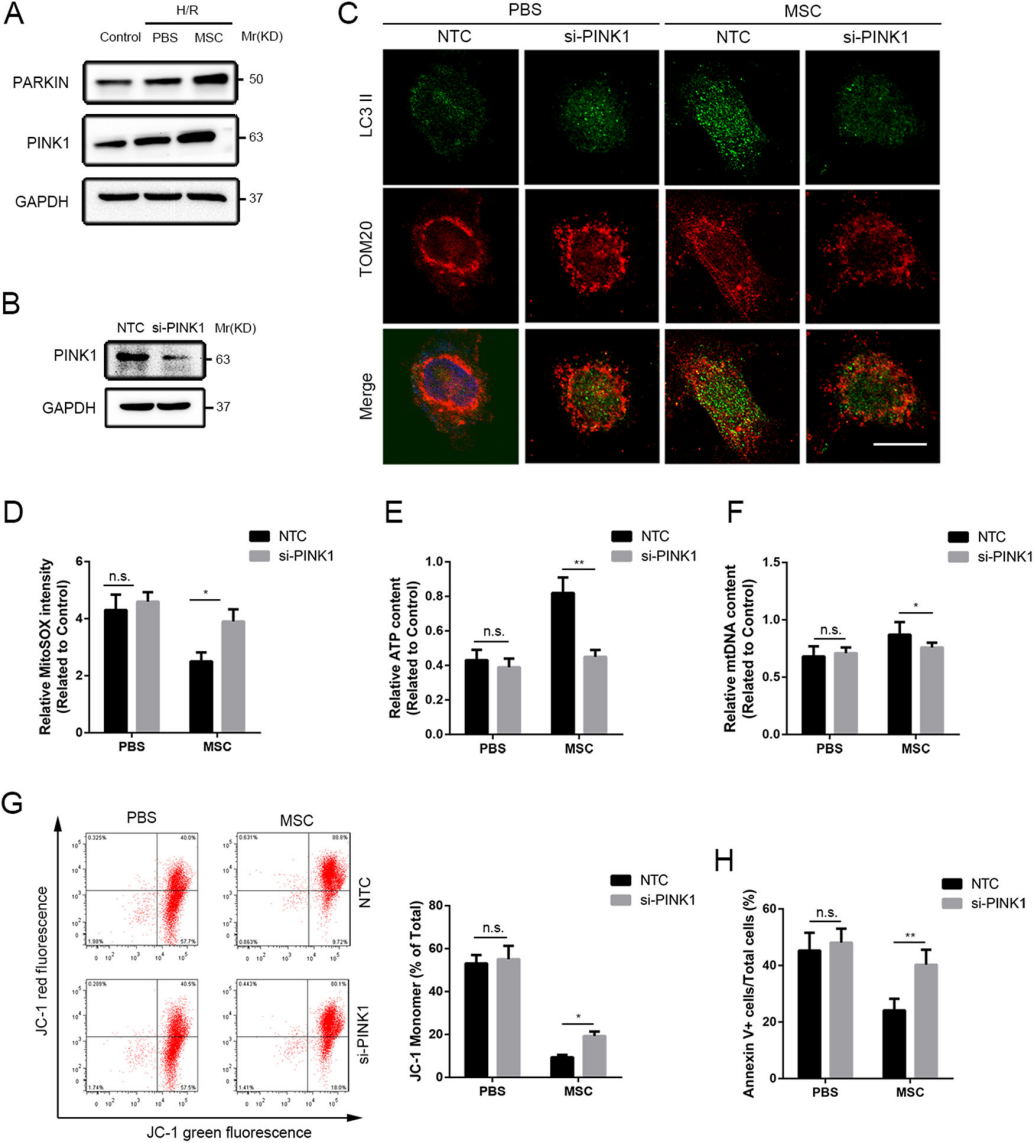
UC-MSC-CM protects L02 hepatocytes exposed to the H/R conditions through a PINK1 mitophagy-dependent mechanism. L02 cells exposed to H/R conditions were divided into four groups: PBS group (treated with PBS), UC-MSCs (treated with UC-MSC-CM), the NTC group (treated with non-silencing scrambled siRNA) and si-PINK1 group (knockdown of PINK1). **a** The expression of Parkin and PINK1 in L02 cells in each group was determined by western blot analysis. **b** To ascertain the effect of the siRNA on PINK1 expression in L02 cells, PINK1 expression was assessed by western blot analysis. **c** To measure the level of mitophagy in L02 cells in each group, the coimmunofluorescence of LC3II (green fluorescence) and TOM20 (red fluorescence) was assessed. Scale bar: 10μm. **d** The level of mtROS generation in each experimental group was detected by MitoSOX Red staining and analyzed by flow cytometry. The data are presented as the means ± SEMs (*n* = 3/group). **e** The level of ATP production in each group was measured. The data are presented as the means ± SEMs (*n* = 3/group). **f** The level of mtDNA in L02 cells in each group was detected. The data are presented as the means ± SEMs (*n* = 3/group). **g** The △Ψm of the inner mitochondrial membrane of L02 cells in each group was detected by the flow cytometric assessment of JC-1 staining. The data are presented as the means ± SEMs (*n* = 3/group). **h** Cell apoptosis was determined through the flow cytometric detection of PI/Annexin V. The data are presented as the means ± SEMs (*n* = 3/group). *p < 0.05, **p < 0.01, ***p < 0.001 (all *p*-values were obtained by one-way ANOVA).

Through an in vitro experiment, we further investigated the mechanism underlying the activation of mitophagy in hepatocytes by UC-MSC-CM during exposure to H/R conditions. The western blot assays showed that expression levels of PINK1 and Parkin were partially enhanced in L02 cells exposed to H/R conditions compared with the control levels, and UC-MSC-CM further increased these changes (Fig. 7a). To clarify the involvement of alterations in PINK1 and Parkin expression in L02 cells during exposure to H/R conditions following UC-MSC-CM treatment, we knocked down PINK1 expression in L02 cells by siRNA using a previously described protocol (Fig. 7b)^33^. Decreases in LC3 expression (green fluorescence) and increases in TOM20 expression (red fluorescence) were observed in coimmunofluorescent photographs of L02 cells exposed to H/R after silencing of PINK1 expression. Furthermore, silencing of PINK1 in L02 cells notably abolished the effect of UC-MSC-CM on upregulation of mitophagy and scavenging of mitochondrial fragments (Fig. 7c). Moreover, the roles of UC-MSC-CM in inhibiting H/R-induced mtROS and mtDNA and restoring ATP generation in L02 cells were attenuated by PINK1 siRNA (Fig. 7d–f). In addition, knockdown of PINK1 expression reversed the stability of △Ψm in L02 cells exposed to H/R conditions and treated with UC-MSC-CM (Fig. 7g). Finally, we detected the viability of L02 cells from each group and found that silencing of PINK1 partially abolished the effect of UC-MSC-CM on inhibiting hepatocellular apoptosis (Fig. 7h).

### UC-MSC-CM-mediated hepatoprotection and PINK1-dependent mitophagy in L02 cells exposed to H/R conditions through AMPKα activation

**Fig. 8:**
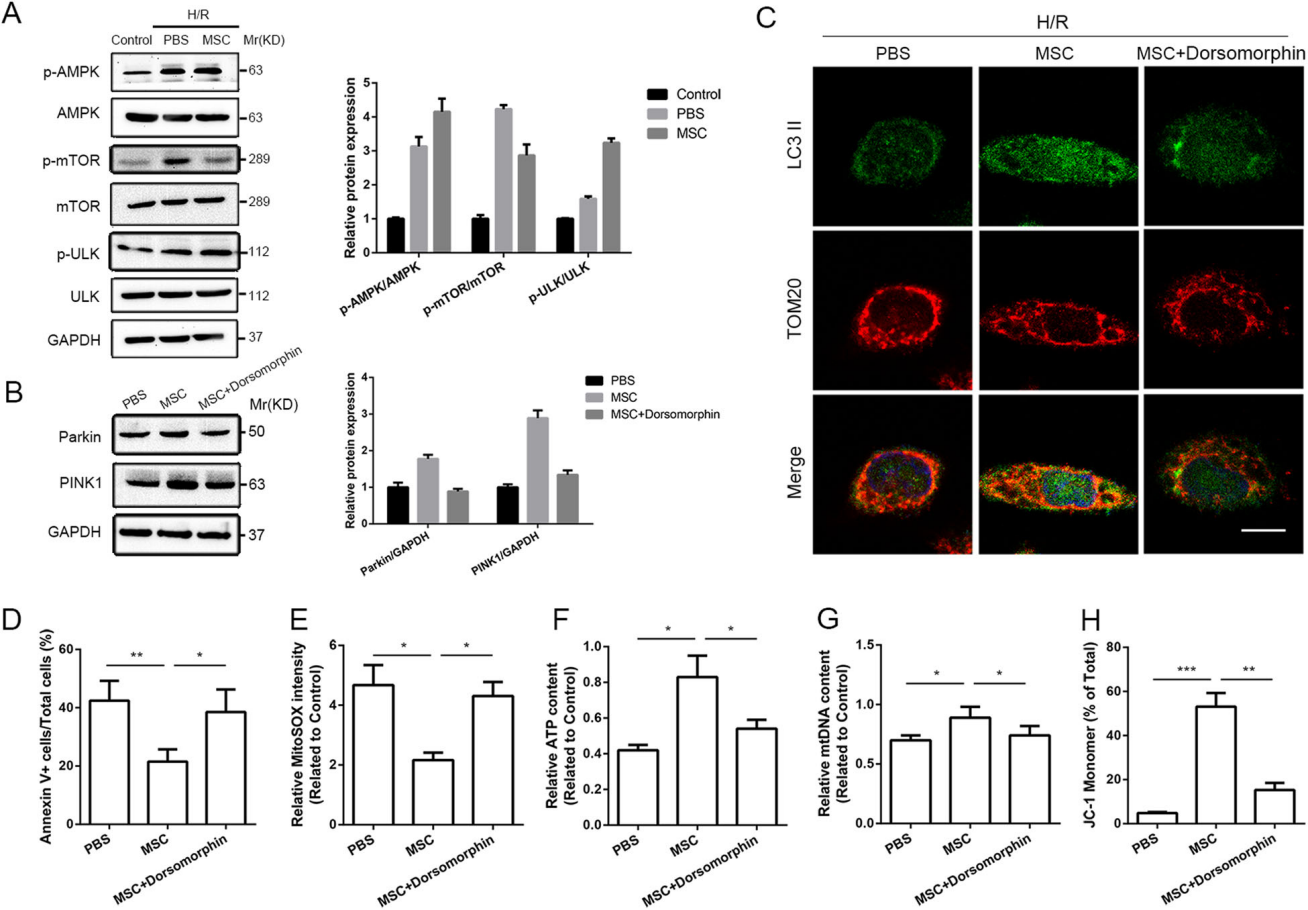
The effect of UC-MSC-CM on enhancing mitophagy in L02 hepatocytes depends on AMPKα activation. L02 cells were divided into four groups: the control group (treated with normal cell culture conditions), PBS group (treated with PBS and exposed to H/R conditions), UC-MSCs (treated with UC-MSC-CM and exposed to H/R conditions) and MSC+ dorsomorphin group (treated with UC-MSC-CM and dorsomorphin, and exposed to H/R conditions). **a** The expression of AMPK, p-AMPK, mTOR, p-mTOR, ULK and p-ULK in L02 cells in each group was determined by western blot analysis. The average intensities of the band in the western blots were quantified using GAPDH as an internal reference. The data are presented as the means ± SEMs (*n* = 3/group). **b** The expression of Parkin and PINK1 in L02 cells in each group was determined by western blot analysis. The average intensities of the band in the western blots were quantified using GAPDH as an internal reference. The data are presented as the means ± SEMs (*n* = 3/group). **c** To measure the level of mitophagy in L02 cells in each group, the coimmunofluorescence of LC3II (green fluorescence) and TOM20 (red fluorescence) was assessed. Scale bar: 10 μm. **d** Cell apoptosis was determined through the flow cytometric detection of PI/Annexin V. The data are presented as the means ± SEMs (*n* = 3/group). **e** The level of mtROS generation in each experimental group was detected by MitoSOX Red staining and analyzed by flow cytometry. The data are presented as the means ± SEMs (*n* = 3/group). **f** The level of ATP production in each group was measured. The data are presented as the means ± SEMs (*n* = 3/group). **g** The level of mtDNA in L02 cells in each group was detected. The data are presented as the means ± SEMs (*n* = 3/group). **h** The △Ψm of the inner mitochondrial membrane of L02 cells in each group was detected through the flow cytometric assessment of JC-1 staining. The data are presented as the means ± SEMs (*n* = 3/group). **g** *p < 0.05, **p < 0.01, ***p < 0.001 (all *p*-values were obtained by one-way ANOVA).

To further confirm the mechanism underlying the induction of PINK1-dependent mitophagy by UC-MSC-CM, alteration in the AMPKα-related pathway in hepatocytes was studied. L02 cells subjected to H/R culture conditions showed decreased expression of p-AMPKα and p-ULK1 and increased expression of p-mTOR in western blot analysis. These changes were notably reversed by UC-MSC-CM treatment (Fig. 8a). We subsequently blocked AMPKα activity in hepatocytes using dorsomorphin, exposed the cells to H/R conditions and then treated the cells with UC-MSC-CM. Western blot assay showed that dorsomorphin partially weakened the roles of UC-MSC-CM in upregulating expression of Parkin and PINK1 in hepatocytes (Fig. 8b). Coimmunofluorescence observation of autophagosomes (LC3II) and mitochondria (TOM20) revealed that the overlap in immunofluorescence was inhibited by AMPKα inactivation, and this finding was compared to the results obtained in L02 cells treated with UC-MSC-CM (Fig. 8c). Moreover, we found that apoptotic ratio was increased in L02 cells treated with combination of UC-MSC-CM and dorsomorphin under H/R conditions (Fig. 8d). Analysis of the changes in mitochondrial quality demonstrated that reduced levels of mtROS production and mtDNA accumulation, increased ATP generation and stabilization of mitochondrial electrochemical gradient obtained with UC-MSC-CM were notably alleviated by addition of dorsomorphin (Fig. 8e–h).

## Discussion

Ample lines of evidence demonstrate protective effects of UC-MSCs in various diseases, but the mechanism through which UC-MSCs attenuate liver I/R injury remains unclear. Our study revealed that administration of UC-MSCs induced hepatocellular mitophagy both in vivo and in vitro, and this effect has a critical role in scavenging of mtROS, reduction of mitochondrial fragmentation and inhibition of cell apoptosis. Conversely, we showed that hepatoprotective effect of UC-MSC-mediated mitophagy was weakened by 3-MA in the I/R injury model and was abolished by knockdown of PINK1 in H/R-treated primary cultured L02 cells. Consistently, exogenous addition of dorsomorphin also attenuated UC-MSC-mediated mitophagy and aggravated degree of liver damage. The data obtained in the present study clearly indicated that AMPKα activation by UC-MSCs contributed to the expression of the mitophagy-related proteins PINK1 and Parkin in hepatocytes. Thus, this study provides the first indication that UC-MSC-mediated mitophagy in hepatocytes protects against ischemic liver injury, which illustrates opportunities for considering PINK1-dependent mitophagy as a potential mechanism for hepatoprotective role of UC-MSCs.

Liver I/R injury is a complex process with multiple stages, including ischemia, early reperfusion and injury phases. ROS overproduction after reperfusion is one of the most important factors for disease progression^34^. Increased oxidative stress leads to activation of several downstream transcription factors and thereby acceleration of the inflammatory response and cell death. Damaged mitochondria induce cytotoxicity by producing superoxide radicals from the abnormal electron transport chain, and mitochondria are also vulnerable to ROS^35,36^. To detect whether the maintenance of mitochondrial quality was due to the inhibition of mtROS production, we used MitoTEMPO, a specific mtROS scavenger^37,38^, for treatment of liver I/R injury. Similar to previous finding reported by Sang et al., we found that MitoTEMPO not only inhibited mtROS accumulation but also mitigated I/R-induced liver damage, as evidenced by a reduction in the level of serum hepatic enzymes and improvement in liver pathology^39^.

Although conventional antioxidants have beneficial roles in improving liver I/R injury, these reagents are not convenient for clinical application, largely because their effects and side effects are not fully understood^40^. UC-MSCs are considered as a promising therapeutic tool for several liver diseases^41–45^. This study also confirmed that UC-MSCs protected both liver tissue and L02 hepatocytes against I/R and H/R injury, as demonstrated by decrease of serum liver enzymes, alleviation of liver injury and reduced inflammatory response. In addition, the current study provided a better complementary mechanism underlying the role of UC-MSCs in protecting against hepatocellular damage under I/R conditions. The results showed that I/R injury led to aggravated oxidative stress, and UC-MSCs notably decreased the MDA level, which is a final product of peroxidation, and enhanced the level of SOD, which has a critical role in clearing ROS. Thus, the findings indicate that UC-MSCs improve the pathological conditions of I/R injury by not only limiting ROS overproduction but also restoring the antioxidant systems. Autophagy has been recognized as an essential and conserved process that affects cell degradation and maintains cell survival in a stress environment^46^. Several studies have revealed that liver I/R injury enhances autophagy^47^. The role of mitophagy in ischemic damage remains controversial. Tang et al.^48^ demonstrated that the activation of PINK1-related mitophagy has a key role in protection against renal ischemia/reperfusion injury, whereas Feng et al.^49^ showed that estrogen exerts cardioprotective effects by reducing mitochondrial dysfunction and inhibiting mitophagy. This study showed that UC-MSCs not only enhanced the autophagic flux, as evidenced by an upregulation of the LC3II/I ratio, but also increased the number of autophagosomes to alleviate hepatocellular damage in vivo. We also investigated the increased loss of mitochondrial fragments accumulation by UC-MSCs treatment in liver I/R injury. The degree of mitochondrial loss and the activity of autophagy was reversed by 3-MA, and this treatment also aggravated the severity of liver damage. The mitophagy-related proteins PINK1 and Parkin have important roles in the mitophagy process and are crucially involved in the maintenance of cell survival. After mitochondrial injury, PINK1 is phosphorylated on depolarized mitochondria, and Parkin is then activated and translocated to mitochondria to initiate mitophagy^50^. A previous study suggested that genipin upregulates hepatocellular mitophagy in a PINK1-dependent manner during I/R injury^20^. We observed a change in the PINK1-dependent signaling pathway in the presence or absence of UC-MSCs and demonstrated that UC-MSC treatment induced pathological improvements accompanied by the upregulation of PINK1 and Parkin expression in liver tissue, particularly in hepatic parenchymal region after I/R injury. Silencing of PINK1 and subsequent inhibition of Parkin activation markedly reversed the protective role of UC-MSCs in hepatocytes and reduced the degradation of dysfunctional mitochondria due to the accumulation of mitochondrial fragments and mtROS.

Consistently, we also detected potential molecular mechanism underlying the effects of UC-MSCs on upregulating mitophagy under I/R conditions. AMPK, a sensor of the inner nutrition and energy status, is composed of α, β and γ subunits^51^. Compelling evidence shows that AMPKα is closely related to the regulation of cell autophagy, proliferation, apoptosis, redox equilibrium and polarity, and AMPK is associated with ULK1 under both physiological and oxidative stress conditions. Activation of the ULK1 complex at Ser555 by AMPKα initiates autophagic vacuole formation. Previous study showed that activation of the AMPK/ULK1 pathway by isoflurane markedly attenuates liver I/R injury and that inhibition of AMPKα phosphorylation increases hepatic damage and oxidative stress in mice with I/R injury^30,52^. Recent studies have demonstrated that activation of AMPKα could upregulate cell mitophagy through its translocation to mitochondria^53^. The activation of AMPKα can enhance PINK1-dependent mitophagy and provide protection against heart failure deterioration^31^. However, the role played by AMPKα activation in the effects of UC-MSCs on inducing hepatocellular mitophagy and controlling mitochondrial quality during I/R injury are unclear. In this study, we performed in vivo and in vitro studies to demonstrate that UC-MSCs protected hepatocytes against I/R injury by upregulating mitophagy through activation of AMPKα. Furthermore, we used dorsomorphin to block AMPKα activation and found that this antagonist could weaken the hepatoprotective effects of UC-MSCs and reverse the increased expression of PINK1 and Parkin in hepatocytes. These results illustrate the key role of UC-MSCs in liver I/R injury through activation of AMPKα, which not only resulted in the direct phosphorylation of PINK1 but also positively influenced Parkin expression through the phosphorylation of ULK1^31,54^. However, further insights are needed to provide sufficient evidence for clinical application.

Collectively, the current study used in vivo and in vitro models to demonstrate the novel mechanisms through which UC-MSCs improve hepatocellular apoptosis during I/R injury. The mechanisms underlying the roles of these cells under I/R conditions might involve the upregulation of mitophagy in hepatocytes through the activation of AMPKα to regulate PINK1 transcription, which leads to inhibition of both mtROS accumulation and aberrant mitochondrial dynamics and eventually results in amelioration of hepatocellular injury and apoptosis (Supplemental Fig. 6). These results further suggest that UC-MSCs might target the maintenance of mitochondrial quality, which is an attractive therapeutic tool for preventing hepatocellular damage in I/R injury.

## Supplementary information

Supplemental information
