# Supplementary material for: MSCs ameliorate hepatocellular apoptosis mediated by PINK1-dependent mitophagy in liver ischemia/reperfusion injury through AMPKα activation
Source: Cell Death Dis. 2020 Apr 20;11(4):256. doi: 10.1038/s41419-020-2424-1 (PMC7171190; doi:10.1038/s41419-020-2424-1)
Supplement: Supplementary file 1 — Supplemental information [file 41419_2020_2424_MOESM1_ESM.docx]

**SUPPLEMENTAL INFORMATION**

**SUPPLEMENTAL FIGURES 1-6**

**
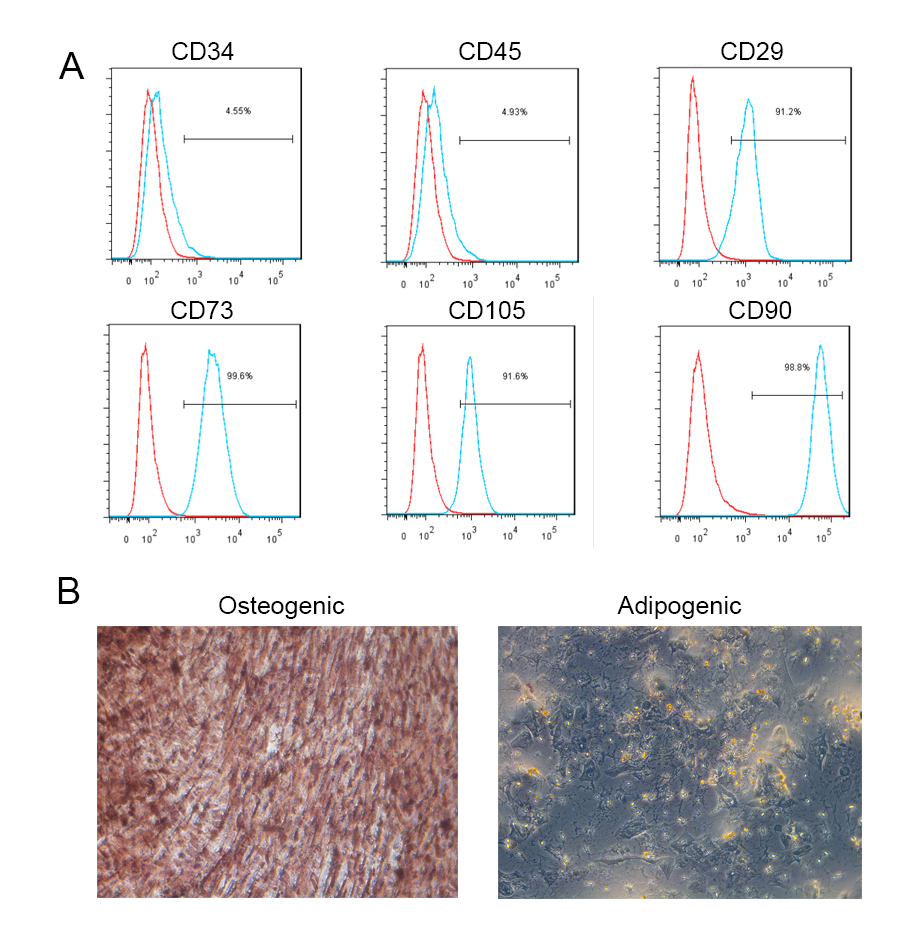
**

**Supplemental figure 1. Isolation and identification of UC-MSCs.**

(A) The cell surface makers were determined by flow cytometric analysis, which were high expression of CD29, CD73, CD105 and CD90, and negative expression of CD34 and CD45. (B) Representative images of UC-MSCs, which differentiate into osteogenesis and adipogenesis.


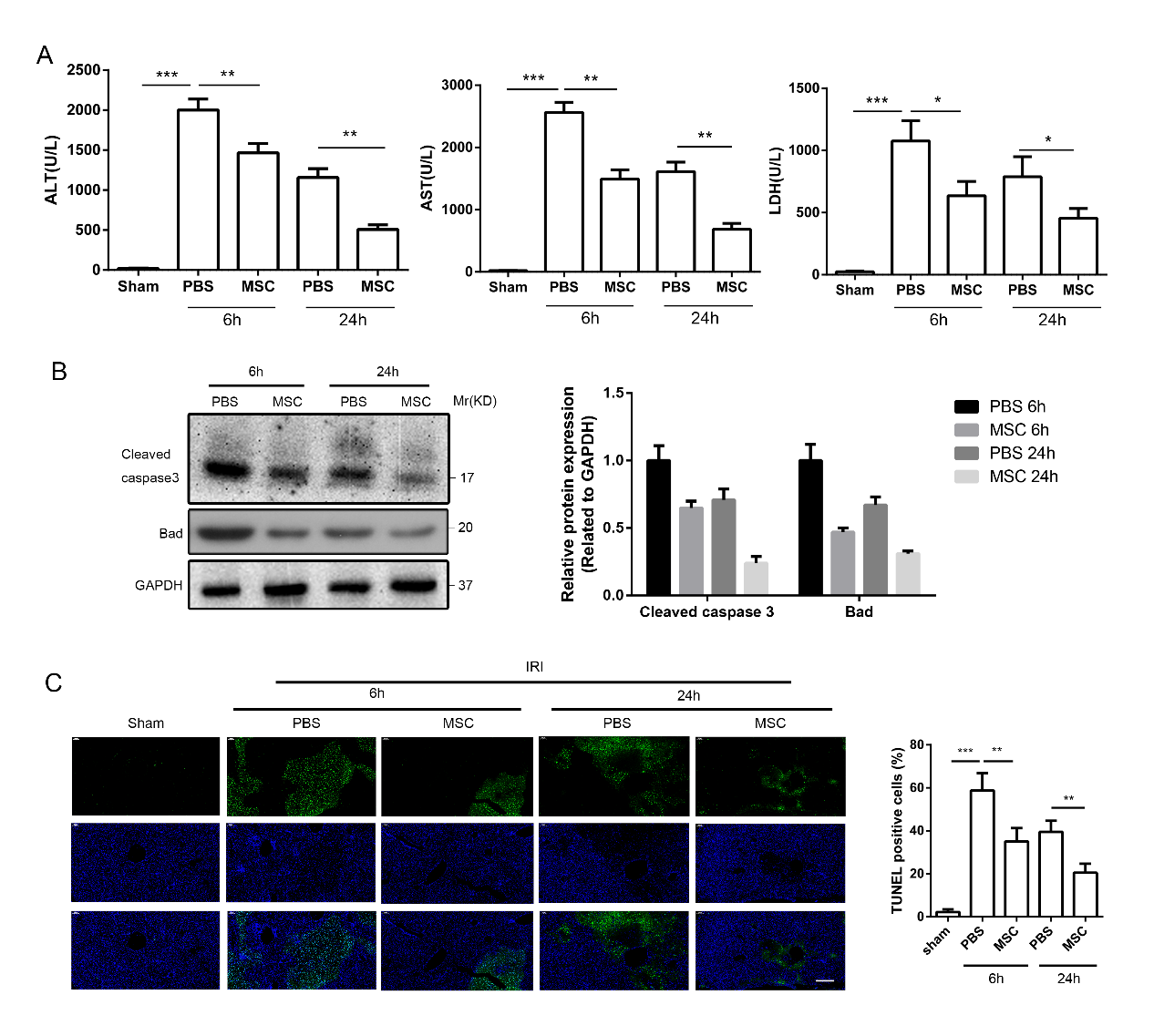


**Supplemental figure 2. Effects of UC-MSCs treatment on improving liver function and inhibiting hepatocellular apoptosis in the IRI mice.**

Mice that underwent hepatic IRI and were treated with UC-MSCs or PBS were sacrificed at 6 h and 24 h after reperfusion. (A) Treatment with UC-MSCs attenuated the levels of ALT, AST and LDH compared with the levels found in the PBS groups. The data are express as the means±SEMs (n=5/group). (B) Western blot assays were performed to detect the level of cleave caspase-3 and Bad in liver tissues from each group. The average intensities of the band in the Western blots were quantified using GAPDH as an internal reference. (C) Representative liver sections of TUNEL staining from each group were obtained (magnification ×100). The data are express as the means±SEMs (n=5/group). *p<0.05, **p<0.01, ***p<0.001 (all p values were obtained by one-way ANOVA).


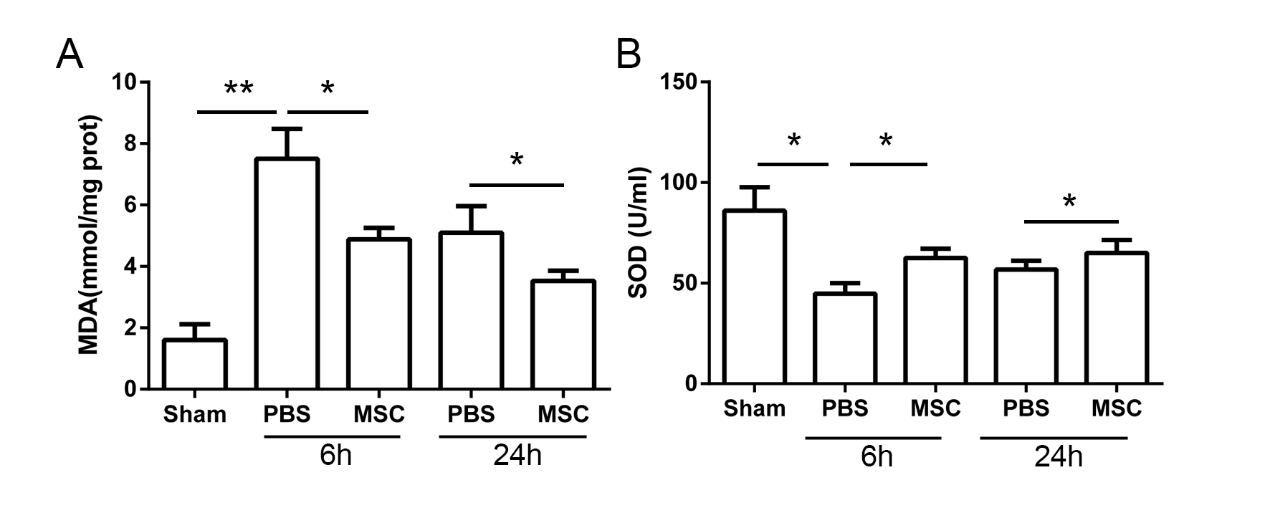


**Supplemental figure 3. Effect of UC-MSCs treatment on improving oxidative stress in the liver of mice with IRI.**

Mice that underwent hepatic IRI and were treated with UC-MSCs or PBS were sacrificed at 6 h and 24 h after reperfusion. (A-B) The levels of MDA and SOD in the liver tissues from each group were detected. The data are express as the means±SEMs (n=5/group). *p<0.05, **p<0.01, ***p<0.001 (all p values were obtained by one-way ANOVA).


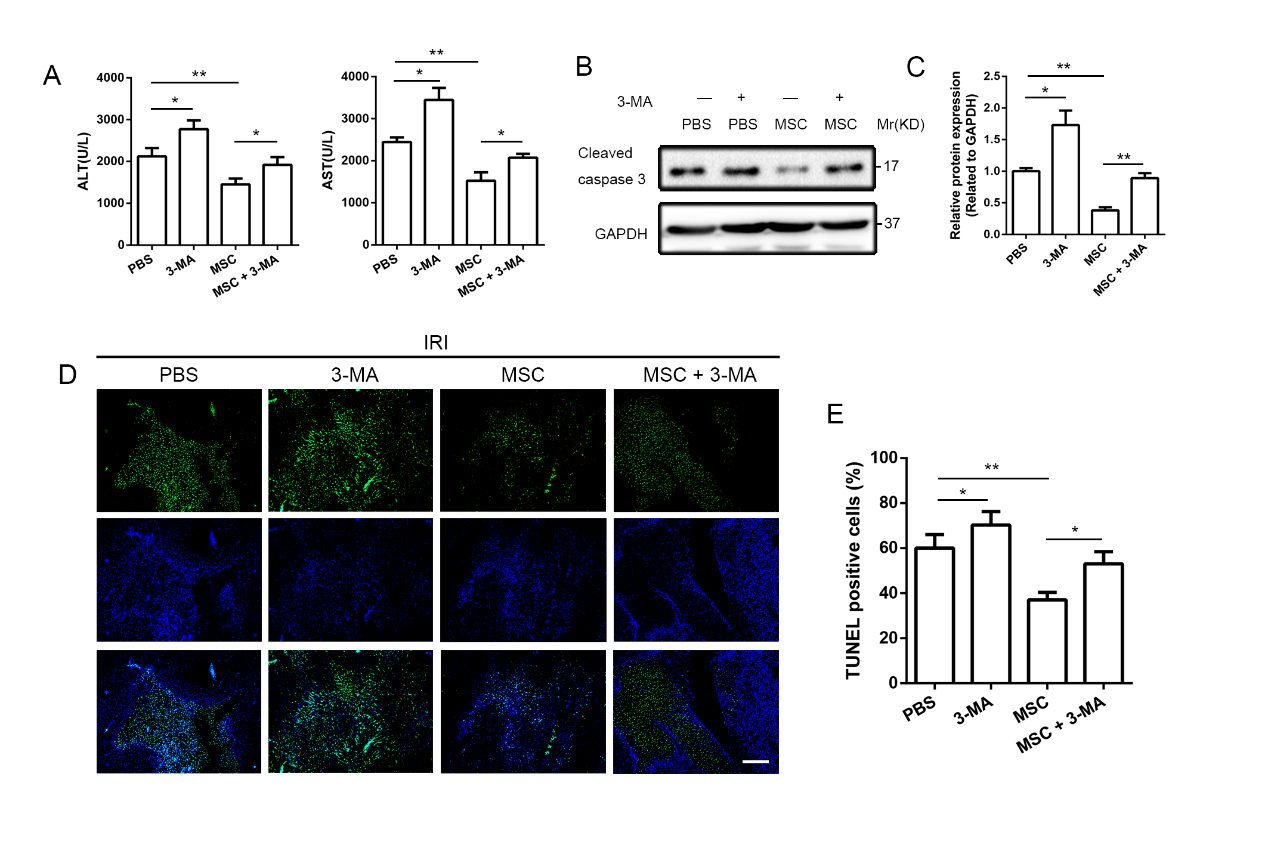


**Supplemental figure 4. 3-MA weakens the effects of UC-MSCs treatment on hepatoprotection and upregulation of autophagy in the liver of mice with IRI.**

Mice with liver IRI were treated with PBS, 3-MA, UC-MSCs or UC-MSCs+3-MA were sacrificed 6 h after reperfusion. (A) The levels of ALT and AST from each group were detected. The data are express as the means±SEMs (n=5/group). (B-C) Western blot assays were performed to detect the level of cleave caspase-3 in liver tissues from each group. The average intensities of the band in the Western blots were quantified using GAPDH as an internal reference. (D-E) Representative liver sections of TUNEL staining from each group were obtained (magnification ×100). The data are express as the means±SEMs (n=5/group). *p<0.05, **p<0.01, ***p<0.001 (all p values were obtained by one-way ANOVA).


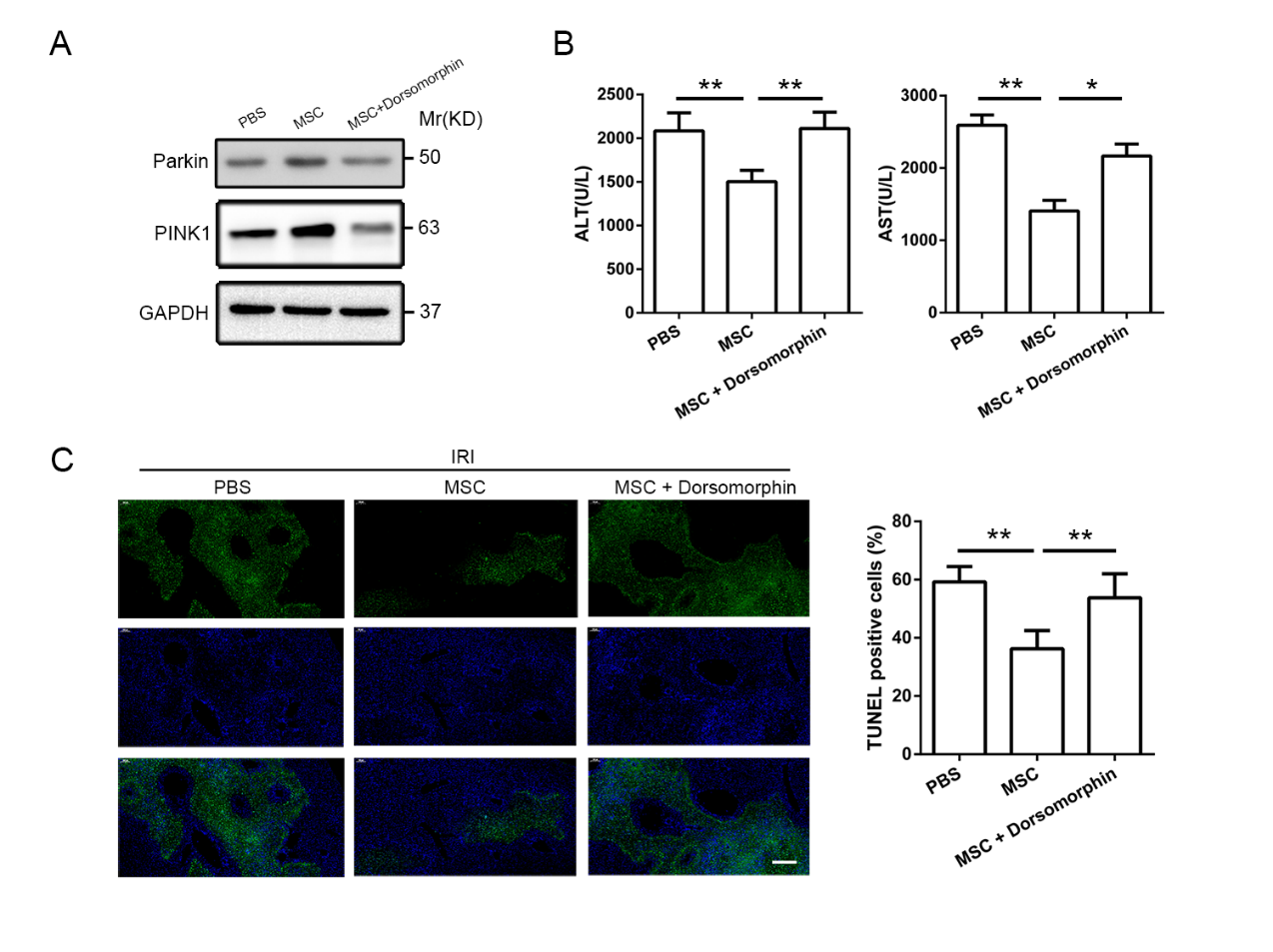


**Supplemental figure 5. Dorsomorphin weakens the effects of UC-MSCs treatment on hepatoprotection in the liver of mice with IRI.**

Mice with liver IRI were treated with PBS, UC-MSCs or UC-MSCs+ Dorsomorphin were sacrificed 6 h after reperfusion. (A) The expression of Parkin and PINK1 in liver tissues was determined by Western blotting. (B) The levels of serum ALT and AST from each group were detected. The data are express as the means±SEMs (n=5/group). (C) Representative liver sections of TUNEL staining from each group were obtained (magnification ×100). The data are express as the means±SEMs (n=5/group). *p<0.05, **p<0.01, ***p<0.001 (all p values were obtained by one-way ANOVA).


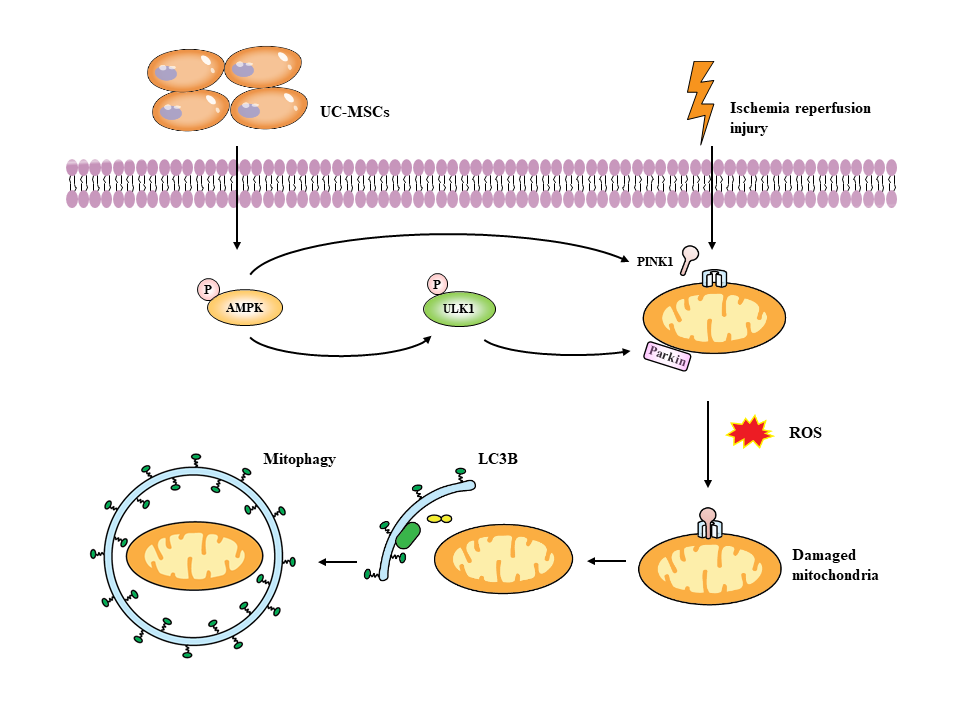


**Supplemental figure 6. Hepatoprotective effect of UC-MSCs.**

Schematic diagram showing the potential molecular mechanisms through which UC-MSCs prevent hepatocellular damage by upregulating mitophagy and maintaining mitochondrial quality after I/R injury.
